# The VanS sensor histidine kinase from type-B vancomycin-resistant enterococci recognizes vancomycin directly

**DOI:** 10.1016/j.jbc.2025.110276

**Published:** 2025-05-22

**Authors:** Lina J. Maciunas, Photis Rotsides, Elizabeth J. D’Lauro, Samantha Brady, Joris Beld, Patrick J. Loll

**Affiliations:** 1 Department of Biochemistry and Molecular Biology, Drexel University College of Medicine, Philadelphia, Pennsylvania, USA; 2 Department of Microbiology and Immunology, Drexel University College of Medicine, Philadelphia, Pennsylvania, USA

**Keywords:** antibiotic resistance, Gram-positive bacteria, histidine kinase, signal transduction, membrane protein, two-component system, vancomycin, VanS

## Abstract

Vancomycin-resistant enterococci (VRE) are high-priority targets for new therapeutic development. In VRE, expression of the resistance phenotype is controlled by the VanRS two-component system, which senses the presence of the antibiotic and responds by initiating transcription of resistance genes. VanS is a transmembrane sensor histidine kinase that is known to detect the antibiotic and then transduce this signal to the VanR transcription factor; however, fundamental questions remain about how exactly VanS senses vancomycin. Here, we focus on a purified VanRS system from one of the most clinically prevalent forms of VRE, type B. We show that in a native-like membrane environment, vancomycin strongly stimulates the autokinase activity of type-B VanS. We additionally demonstrate that this effect is mediated by a direct physical interaction between the antibiotic and the VanS periplasmic domain. This represents the first time that a direct sensing mechanism has been confirmed for any VanS protein from a human pathogen.

Antibiotic resistance is a global health problem. Pathogenic bacteria are becoming resistant to currently available antibiotics at an alarming rate, while the development of new antibiotics remains slow (1). Important examples of antibiotic-resistant bacteria include the vancomycin-resistant enterococci (VRE), which have been identified by the World Health Organization as top priorities for new therapeutic development (2, 3). VRE are among the so-called ESKAPE pathogens; these organisms are leading causes of hospital-acquired infections, and few therapeutic options remain for their treatment (4, 5).

Ten different VRE genotypes are currently recognized, and are distributed across multiple species of enterococci. These resistance types are denoted as types A, B, C, D, E, G, L, M, N, and P (also known as VanA, VanB, VanC, and so on (6)); currently, the two types most commonly associated with human disease are types A and B (7). In all cases, VRE evade vancomycin’s antibiotic action by remodeling their cell wall, preventing the antibiotic from binding its target (8). This mode of resistance requires the acquisition of five key genes, *vanRSHAX,* which are necessary and sufficient to confer vancomycin resistance (9). VanHAX are enzymes that remodel vancomycin’s cell-wall target, and their expression is controlled by a two-component system comprising VanR and VanS. VanR is a transcription factor that is activated by phosphorylation; VanS is a membrane-bound sensor histidine kinase that detects vancomycin and modulates the phosphorylation state of VanR accordingly (10).

No structure is currently known for any full-length VanS protein. However, predictions from sources such as AlphaFold, along with structural information from other histidine kinases, suggest that VanS proteins consist of four structural domains: a sensor domain, a membrane-proximal domain, a dimerization-and-histidine phosphotransfer (DHp) domain, and a catalytic and ATP-binding (CA) domain (11, 12) (Fig. 1*A*). The sensor domain is predicted to contain two transmembrane helices flanking a periplasmic region (13, 14), and is likely responsible for recognizing the presence of vancomycin. When VanS senses vancomycin, the signal is transduced to the cytoplasmic domains, which coordinate an enzymatic response involving autophosphorylation on a conserved histidine residue, followed by transfer of the phosphoryl group to VanR (9, 15). Once phosphorylated, VanR initiates transcription of the resistance genes (16). In the absence of an activating signal, VanS can dephosphorylate VanR, terminating the signaling cascade (17, 18). Interrupting this signaling process represents an obvious therapeutic avenue that could salvage the utility of the antibiotic. This goal could be achieved by inhibiting autophosphorylation and phosphotransfer, and/or by stimulating VanR dephosphorylation.

**Figure 1:**
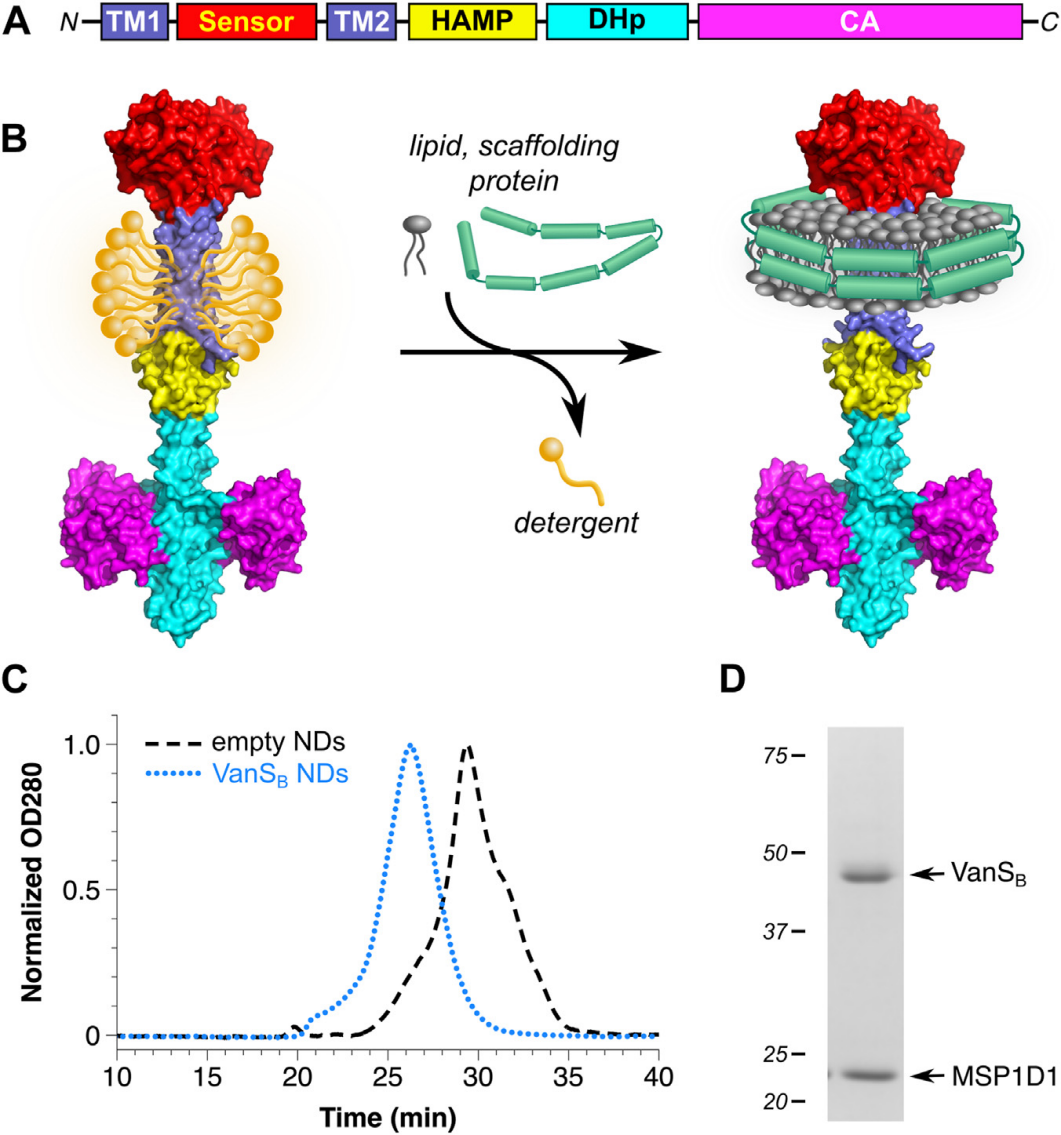
**Reconstitution of VanS_B_ into nanodiscs (NDs)**. *A*, schematic representation of the typical VanS architecture. Two transmembrane helices (TM1 and TM2) flank a periplasmic sensor domain, the length of which varies significantly among different VanS proteins. The cytoplasmic portion of the protein contains a membrane-proximal region, which typically forms a HAMP domain, followed by the DHp (dimerization and histidine phosphotransfer) and CA (catalytic and ATP-binding) domains. *B*, for ND formation, detergent-solubilized protein is incubated with lipids and scaffolding protein, after which detergent is removed, triggering the spontaneous formation of protein-belted lipid discs around the hydrophobic face of the protein. The protein model shown represents the structure of full-length VanS_B_, as predicted by AlphaFold; domain colors match those found in *panel (A)* (81). *C*, size-exclusion chromatograms reveal that NDs containing VanS_B_ elute earlier than empty NDs formed under identical conditions, indicating size increases consistent with the incorporation of the VanS protein. *D*, coomassie-stained denaturing SDS-PAGE gels showing a purified VanS_B_ ND preparation indicating that this preparation contains approximately similar amounts of VanS_B_ and the scaffolding protein MSP1D1.

The largest gap in our understanding of vancomycin resistance centers on how VanS recognizes vancomycin. In principle, VanS could detect vancomycin either directly, *via* a physical interaction with the antibiotic, or indirectly, by sensing a downstream consequence of vancomycin’s action. Multiple different VanS proteins have been studied, including proteins from VRE and homologs from nonenterococcal species, and arguments have been advanced in favor of both models. Chimeric-protein studies, photolabeling, and spectroscopic experiments have all provided data that support a direct-interaction model (19, 20, 21, 22, 23, 24); in contrast, an indirect model is supported by the observation that some VanS proteins can be promiscuously activated by many different inhibitors of cell-wall biosynthesis (based on the reasoning that a single binding site on VanS would be unlikely to recognize a wide array of structurally dissimilar effectors) (25, 26, 27, 28). Importantly, the direct and indirect models are not necessarily mutually exclusive, nor must a single model apply to all VanS proteins. Thus, considerable uncertainty surrounds the molecular mechanism by which VanS recognizes and transduces the vancomycin signal.

To elucidate this process, we have chosen to examine vancomycin recognition in type-B VRE, focusing on the VanS protein from these organisms (denoted herein as VanS_B_). We purified full-length VanS_B_ and inserted it into nanodiscs (NDs). This approach provides the molecular insights gained by reconstituting a signaling system from purified components, while simultaneously maintaining a native-like membrane environment. We investigated the relationship of vancomycin binding to enzymatic activity, showing that vancomycin markedly stimulates VanS_B_ activity, and further demonstrated a direct physical interaction between the VanS_B_ sensor domain and vancomycin. These results link activity changes with direct binding of the antibiotic for the first time for any VanS protein from a VRE pathogen.

## Results

### Reconstitution of VanS into NDs

Membrane proteins are typically purified in detergent-solubilized form, but detergents can reduce stability and/or block access to interaction sites (29, 30, 31). The latter effect is of particular concern in the VanRS system, since it has been suggested that vancomycin interacts with VanS at a site directly adjacent to the membrane (22). To provide a more native-like environment for VanS_B_, we reconstituted it into NDs, lipid-bilayer discs belted by an amphipathic scaffolding protein (32, 33). VanS_B_ NDs were created by mixing detergent-solubilized protein with *Escherichia coli* lipids and the scaffolding protein MSP1D1, after which detergent was removed, allowing the NDs to spontaneously assemble (Fig. 1*B*). VanS_B_-containing NDs were separated from empty NDs by capturing the His-tagged VanS_B_ protein *via* nickel-affinity chromatography, after which aggregated material was removed on a size-exclusion column. Reinjection of the purified material gave symmetrical peaks eluting significantly earlier than empty NDs (Fig. 1*C*). SDS-PAGE analysis revealed that VanS_B_ and MSP1D1 are present in the NDs in similar amounts (Fig. 1*D*). Each ND is known to contain two copies of the MSP1D1 scaffolding protein (33), and thus approximately two copies of VanS_B_ are also present. Since sensor histidine kinases typically form obligate homodimers (34), this suggests that each ND may contain a single homodimer.

Only a small number of sensor histidine kinases have been reconstituted into NDs (35, 36, 37, 38, 39, 40, 41, 42), and this is the first report of this approach being applied to any VanS protein. These reconstituted VanS_B_ protein preparations provide the foundation for the *in-vitro* experiments described below, and allow us to avoid the complications associated with the use of detergent.

### VanS_B_ is active after reconstitution into NDs

**Figure 2:**
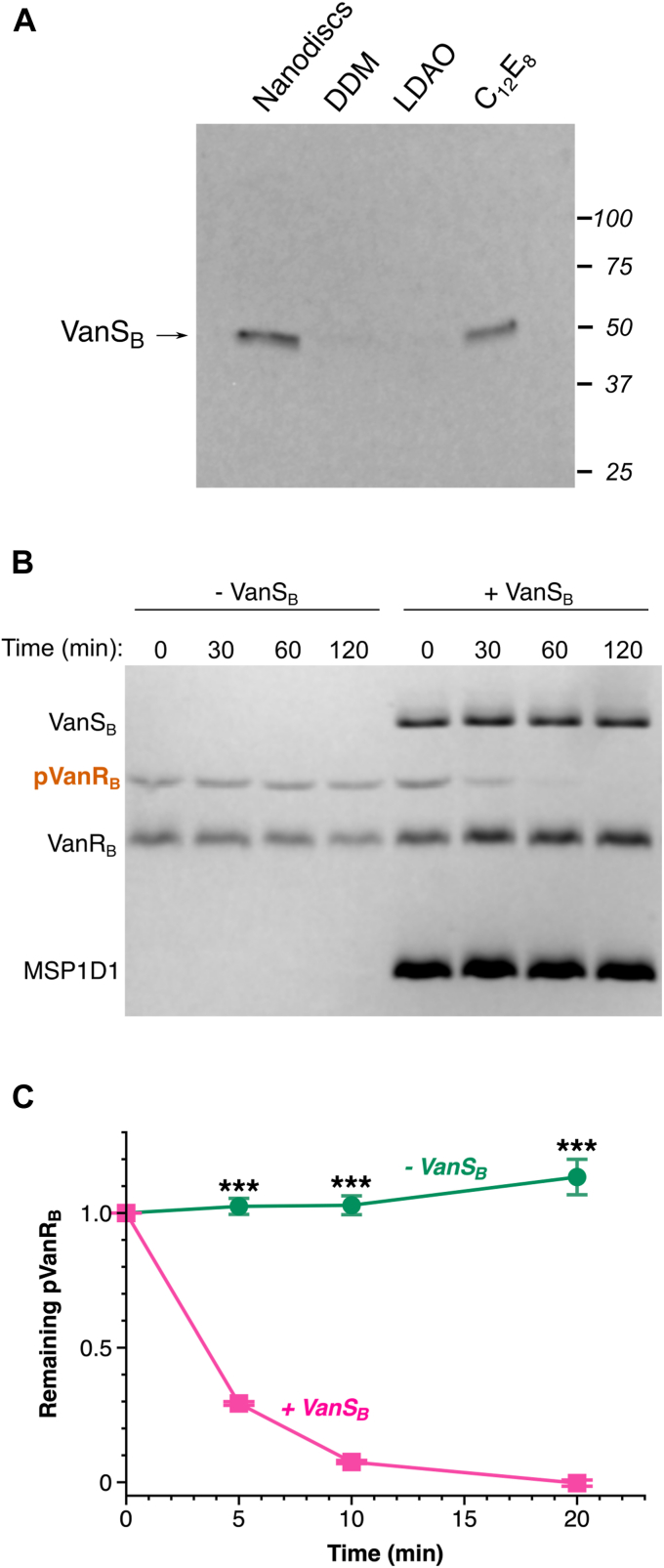
**Enzymatic activities of purified VanS_B_**. *A*, VanS_B_ activity is strongly sensitive to the membrane-mimetic environment. In each reaction, 0.3 μM VanS_B_ was incubated with 1 mM ATPγS for 30 min. The thiophosphohistidine was then alkylated with *p*-nitrobenzyl mesylate (PNBM), which was subsequently detected by Western blot. Equal amounts of protein are loaded in each lane. VanS_B_ shows very little activity in the presence of the detergents DDM and LDAO, and modest activity in C_12_E_8_ and nanodiscs. *B* and *C*, dephosphorylation activity of VanS_B_. Phosphorylated VanR_B_ was incubated ± VanS_B_-containing nanodiscs, and the time-dependent decrease in phosphorylation was monitored using Phos-tag gels. *Panel (B)* shows representative gel, and *panel (C)* shows the quantitation (*n* = 3). *Asterisks* represent *p*-values calculated using an unpaired two-tailed *t* test; ∗∗∗, *p*-value < 0.0001. This experiment illustrates the high intrinsic stability of phospho-VanR_B_ (17), consistent with the observation that VanR_B_ can be isolated in a partially phosphorylated form after expression in *Escherichia coli* (Fig. S10). DDM, *N*-dodecyl β-d-maltoside; LDAO, lauryl dimethyl amine oxide.

Sensor histidine kinases autophosphorylate in response to signals; they also modulate the phosphorylation states of their response regulators, transferring their phosphoryl group to the response regulator. Some sensor histidine kinases exert additional control *via* a phosphatase activity that dephosphorylates their response regulators in the absence of a signal (34). These three activities reside in the cytosolic domains of the kinases, and are regulated by the transmembrane and sensor regions in response to stimulus detection. In the case of VanS_B_, a construct containing only the cytosolic domain has been shown to display all three enzymatic activities (17). To examine the behavior of full-length VanS_B_ in a ND environment, we first focused on the autophosphorylation function. We monitored activity using a modified Western blot assay (Fig. 2*A*), in which VanS_B_ was allowed to autophosphorylate with ATPγS, after which the thiophosphohistidine was alkylated and detected by antibody (43). VanS_B_ is strongly sensitive to the specific membrane mimetic used, a trait shared with VanS_A_, the VanS protein found in type-A VRE (44). Specifically, VanS_B_ shows essentially no activity in the presence of the detergents *N*-dodecyl β-d-maltopyranoside (DDM) or lauryl dimethyl amine oxide (LDAO), and modest activity in octaethylene glycol monododecyl ether (C_12_E_8_). The ND preparation of VanS_B_ is autokinase-active, validating the use of this approach. Interestingly, however, reconstitution of VanS_B_ into NDs leads to only modest basal levels of autokinase activity, failing to surpass the levels seen in the presence of C_12_E_8_. To ensure that the observed activity is specific, we prepared two mutants expected to be autokinase-deficient, namely H233A (in which the histidine that accepts the phosphoryl group is removed) and N349A (in which a catalytically important residue in the so-called N-box region is removed (45)). Neither mutant showed a detectable level of autophosphorylation activity in the autophosphorylation assay (Fig. S1).

We next probed dephosphorylation activity, incubating VanS_B_ with the phosphorylated form of its cognate VanR protein and quantifying the time-dependent decrease in phospho-VanR using Phos-tag gels (46, 47). As anticipated, VanS_B_ was found to catalyze the dephosphorylation of VanR_B_ (Fig. 2, *B* and *C*). Finally, we confirmed that VanS_B_ is capable of phosphotransfer to its cognate VanR_B_ partner (Fig. S2*A*), and that this process is specific, since no phosphotransfer is observed when using a VanR_B_ mutant that lacks the phosphoryl-accepting aspartate residue (Fig. S2*B*). Taken together, these experiments demonstrate that our reconstituted ND preparation of VanS_B_ possesses all three relevant enzymatic activities.

### Vancomycin’s effect on VanS_B_ activity

**Figure 3:**
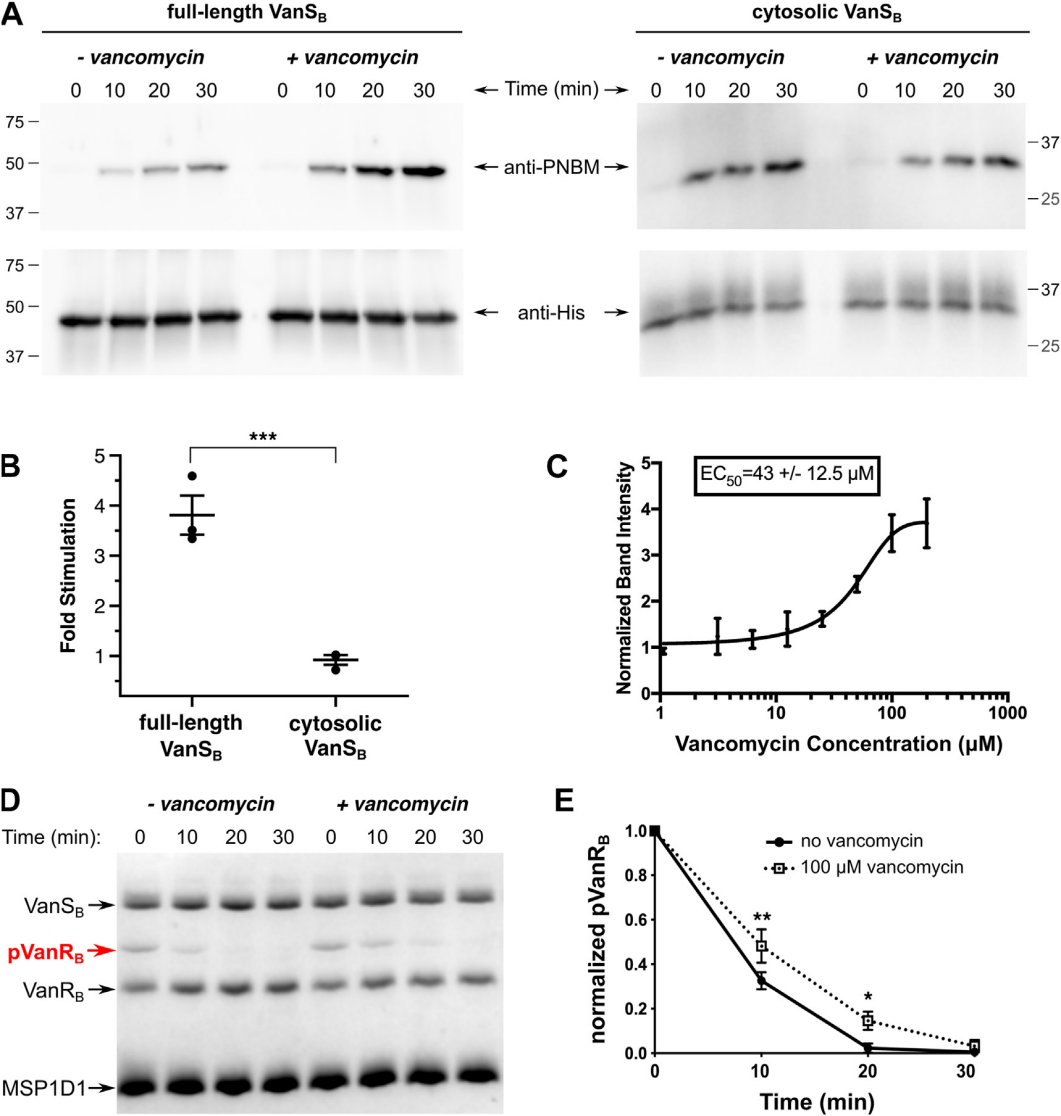
**Effect of vancomycin on VanS_B_ activities***. A*, representative Western blots showing an autophosphorylation time course for full-length VanS_B_ (reconstituted in nanodiscs) and its cytosolic domain, ± 100 μM vancomycin. Positions of molecular-weight markers are indicated. *B*, quantitation of vancomycin stimulation of autophosphorylation activity (*n* = 3). Shown is the fold increase in autophosphorylation levels (relative to no antibiotic) after 30-min treatment with 100 μM vancomycin. ∗∗∗, *p*-value < 0.005. *C*, dose dependence for vancomycin stimulation of VanS_B_ autophosphorylation activity, calculated after 30-min treatment with the antibiotic. *D*, a representative Phos-tag gel demonstrating that 100 μM vancomycin has a modest inhibitory effect on the dephosphorylation activity of VanS_B_. *E*, quantitation of three dephosphorylation experiments for VanS_B_. ∗, *p*-value < 0.05; ∗∗, *p*-value < 0.01. Significance was evaluated using unpaired two-tailed *t*-tests.

We hypothesized that if vancomycin directly activates VanS_B_, then the antibiotic should increase the enzyme’s autokinase activity and/or decrease its phosphatase activity; either effect would tend to increase cellular levels of phospho-VanR_B_. We first investigated the effect of vancomycin on the autokinase activity of VanS_B_, and found that it stimulated autophosphorylation in a concentration-dependent manner (Fig. 3, *A*–*C*). We then showed that this stimulatory effect was also observed using ^32^P-ATP as a kinase substrate, confirming that this result was not unique to ATPγS (Fig. S3). In addition, we prepared a VanS_B_ construct lacking the C-terminal His-tag and demonstrated that its autophosphorylation activity is also stimulated by vancomycin, to a similar extent as that seen in the tagged protein, confirming that the affinity tag is not interfering with the protein’s function (Fig. S4).

When VanS_B_ is embedded in a ND, both its extracellular and intracellular domains will be accessible to a water-soluble molecule such as vancomycin; however, in the native cellular environment, only the periplasmic domain will be accessible to the antibiotic. Therefore, any effect that vancomycin has on the enzyme’s activity should be mediated through the periplasmic sensor domain, and not through any of the cytosolic domains. To confirm the absence of any such nonspecific effects, we purified a cytosolic VanS_B_ construct lacking the sensor domain and showed that vancomycin has no effect on its autophosphorylation activity (Fig. 3*B*). Thus, the stimulatory effect of vancomycin is specific to the full-length VanS_B_ protein.

We also tested whether vancomycin influences the dephosphorylation activity of VanS_B_, and found that it slightly decreased the enzyme’s dephosphorylation of VanR_B_ (Fig. 3, *D* and *E*). Finally, we assessed whether vancomycin affects the rate of phosphotransfer from VanS_B_ to VanR_B_. This reaction is too fast to easily monitor using radioisotope labeling, but within the time regime that is experimentally accessible, we observed no effect of vancomycin on the rate of phosphotransfer (Fig. S2A). This is perhaps not surprising, since the catalytic machinery mediating phosphotransfer typically resides primarily in the response regulator, rather than the sensor kinase (48).

In summary, vancomycin increases VanS_B_’s autophosphorylation activity, modestly decreases its phosphatase activity, and has no effect on the phosphotransferase reaction. Taken together, these effects are consistent with vancomycin directly stimulating VanRS signaling in type-B VRE by elevating levels of phospho-VanS_B_, which will lead in turn to increased amounts of phospho-VanR_B_.

### Vancomycin binds to the periplasmic sensor domain of VanS_B_

**Figure 4:**
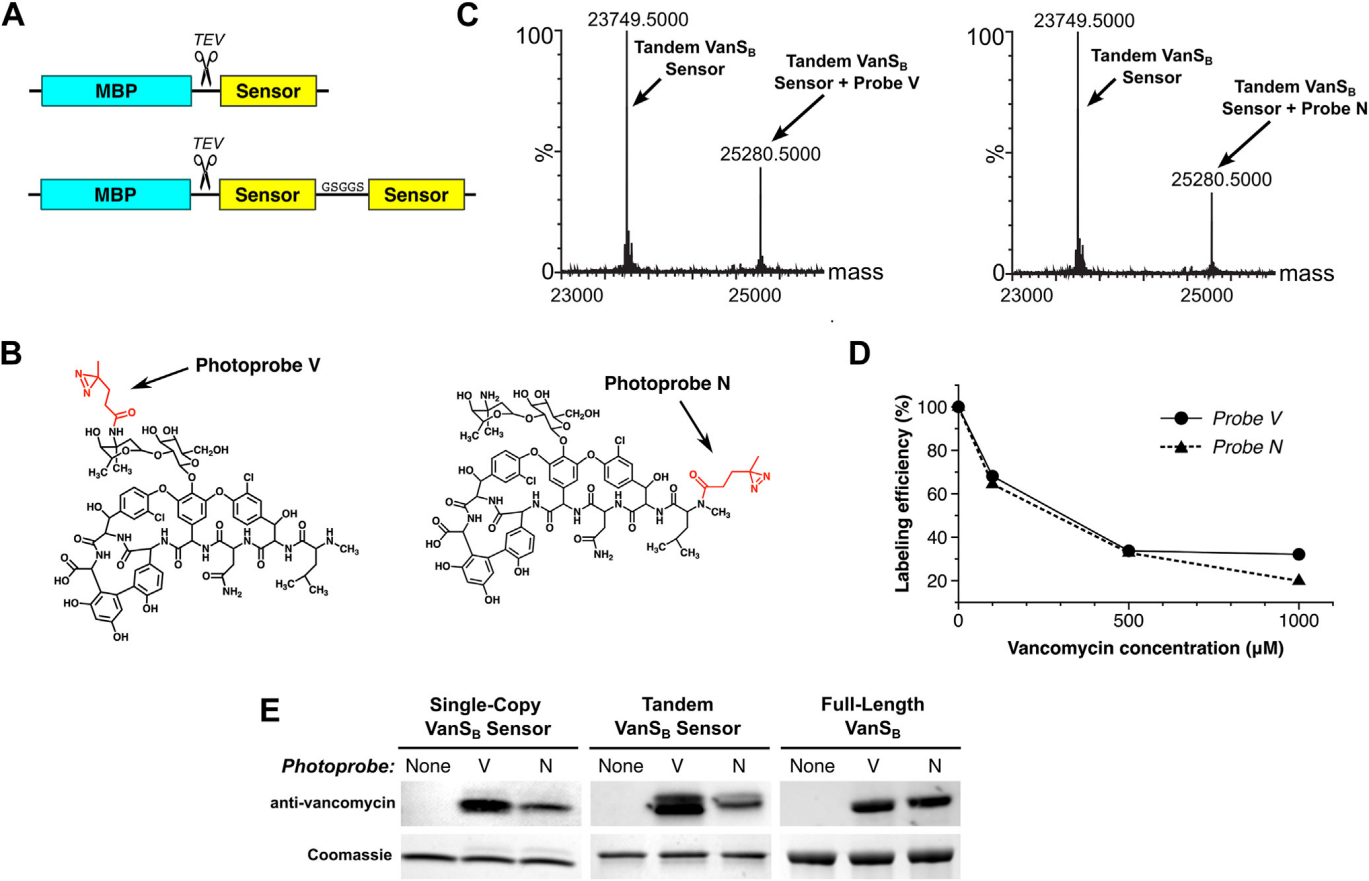
**The VanS_B_ periplasmic sensor domain can be labeled with vancomycin-based photoprobes**. *A*, the VanS_B_ periplasmic sensor domain (residues 31–132) was expressed as a cleavable fusion with MBP, in both single-copy and tandem forms, and purified to homogeneity (Fig. S5). *B*, two photoprobes were used, in which a photo-active diazirine was attached to either the antibiotic’s vancosamine sugar (Photoprobe V) or its N terminus (Photoprobe N). *C*, mass spectrometric evidence that both photoprobes label the purified tandem sensor-domain construct. *D*, vancomycin competes with both photoprobes for binding to the tandem sensor domain. Labeling efficiency was calculated from the mass spectrometric data, with efficiency expressed as the relative peak height for the photolabeled species as compared to that of the unlabeled species. *E*, confirmation of photolabeling using an anti-vancomycin Western-blot assay, showing labeling of both the isolated single-copy and tandem sensor domains, as well as full-length VanS_B_. Coomassie-stained gels serve as loading controls. Photoprobe N typically labels VanS_B_ constructs less efficiently than Photoprobe V, as judged by both Western blotting and mass spectrometry (see *panel (C)*); this may reflect differences in the diazirine group’s proximity to the protein, as determined by the conformation of the bound antibiotic.

Vancomycin’s effect on the enzymatic activity of purified VanS_B_ suggested that the antibiotic might bind directly to the enzyme. We reasoned that the likely site of binding is the protein’s periplasmic region, as it is the only portion of the protein accessible from the outside of the cell. The VanS_B_ periplasmic domain contains roughly 100 residues, and it is predicted to adopt a PAS-domain fold (Fig. S5) (49, 50). To test whether the isolated periplasmic domain is capable of binding the antibiotic, we prepared expression constructs for the appropriate region, corresponding to VanS_B_ residues 31 to 132. We produced two constructs, the first encoding a single copy of the periplasmic sensor, and the second containing two copies of the sensor in tandem, separated by a short linker (Fig. 4*A*). The latter construct was designed to encourage dimerization, since full-length sensor histidine kinases are thought to be obligate dimers (34). Both proteins were purified to homogeneity (Fig. S5*A*).

We first probed antibiotic binding to the periplasmic-domain constructs using two novel photoaffinity probes, in which a diazirine photolabel is attached at either end of the vancomycin molecule (51). Specifically, the photolabel is attached to either the antibiotic’s vancosamine sugar (Probe V) or its N terminus (Probe N; Fig. 4*B*). Excitation of the diazirine with ultraviolet light gives rise to a reactive carbene species, which can react with nearby target molecules to form a covalent bond between the photoprobe and the target (52). The lifetime of the carbene is very short, ensuring that it will only label nearby molecules. If the photoprobe is bound to a protein, that protein can be labeled; otherwise, the carbene will be quenched by water (53). Both photoprobes efficiently labeled the single-copy and tandem VanS_B_ sensor proteins, as demonstrated by mass spectrometry and anti-vancomycin Western blotting (Fig. 4, *C* and *E*). No evidence was seen for double labeling of the tandem sensor domain, even though in principle it can contain two distinct ligand-binding sites (54); however, a covalent dimer is not required for the interaction, since photolabeling was also observed for the single-copy construct. Unlabeled vancomycin competed effectively with both photoprobes, indicating that the protein-photoprobe interaction is specific (Fig. 4*D*). Notably, both photoprobes also label the full-length VanS_B_ protein, indicating that the observed interaction with the sensor domain is not an artifact caused by removing the periplasmic domain from the context of the intact protein molecule. Overall, these results provided strong evidence of a specific, direct interaction between vancomycin and the VanS_B_ protein.

**Figure 5:**
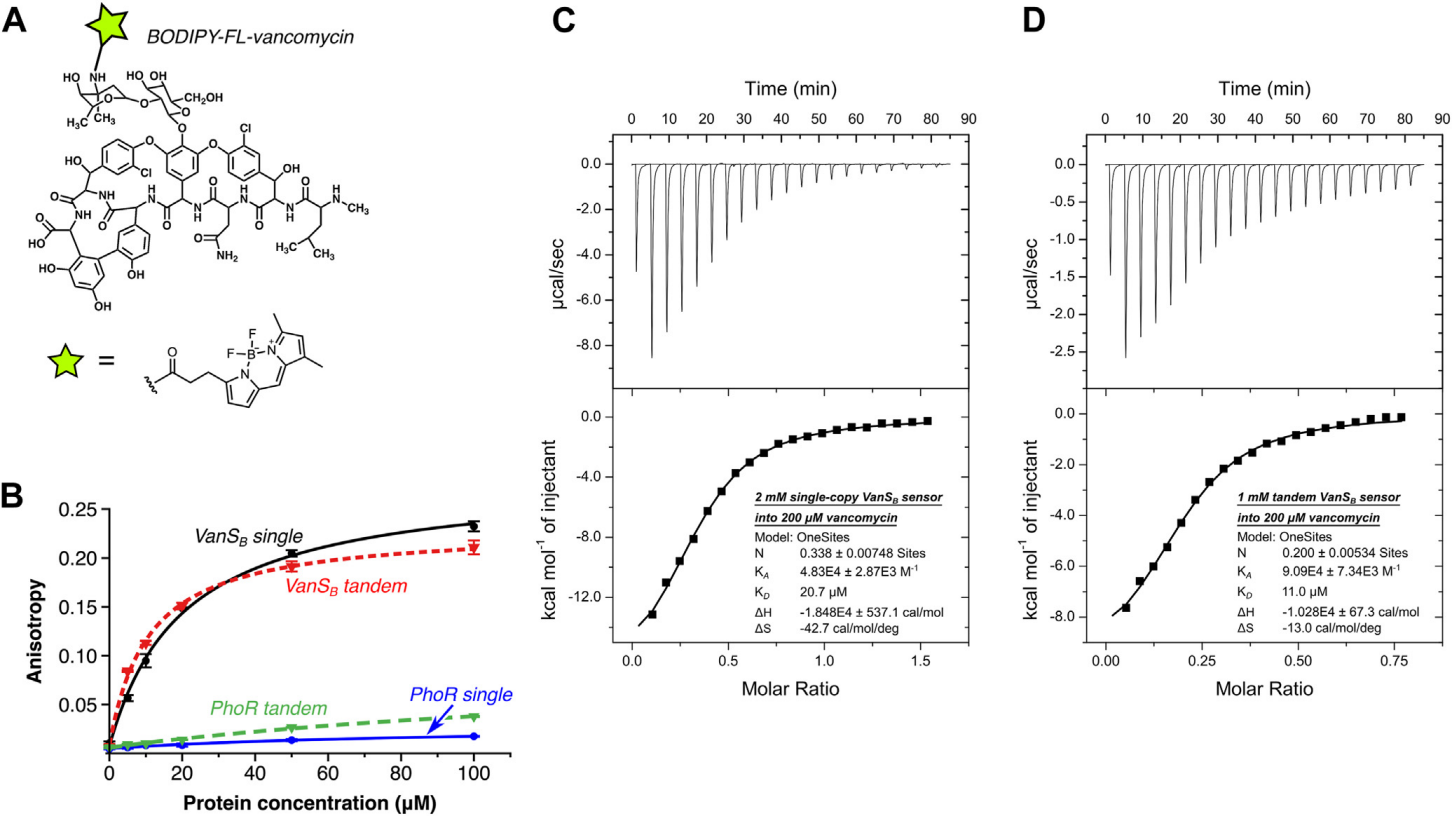
**Vancomycin binds directly to the periplasmic sensor domain of VanS_B_**. *A*, fluorescently labeled vancomycin derivative used for fluorescence anisotropy measurements. *B*, BODIPY-FL-vancomycin binds to both the single and tandem sensor-domain constructs of VanS_B_. In contrast, single and tandem constructs of the PhoR negative control fail to bind the antibiotic. *C* and *D*, isothermal titration calorimetry confirms vancomycin binding by the single-copy *(C)* and tandem *(D)* VanS_B_ sensor-domain constructs. The related glycopeptide antibiotic teicoplanin fails to bind to the sensor domain (Fig. S7), which is consistent with the known behavior of type-B VRE. VRE, vancomycin-resistant enterococci.

We next sought to quantify this binding interaction, using fluorescence anisotropy to assess recognition of BODIPY-FL-labeled vancomycin (Fig. 5). The single-copy VanS_B_ sensor domain was found to bind to the labeled vancomycin with a dissociation constant (*K*_D_) of 20.3 μM, while the tandem sensor bound with a *K*_D_ of 10.7 μM; the fact that the single-domain *K*_D_ value is almost exactly twice that of the tandem domain suggests that there is no cooperativity between the two domains, at least in the constructs used. We then generated negative controls, creating single- and tandem-domain versions of the periplasmic sensor domain from the *Bacillus subtilis* histidine kinase PhoR. The PhoR sensor domain adopts a PAS fold (55), similar to that predicted for the VanS_B_ periplasmic domain. However, PhoR is not expected to bind to vancomycin, and indeed, neither PhoR construct bound to BODIPY-FL-vancomycin (Fig. 5*B*). Next, to test whether the binding observed in the anisotropy experiment was fluorophore-dependent, we created a new fluorescent probe by labeling vancomycin with AlexaFluor488, instead of BODIPY-FL (Fig. S6). The fluorophores are attached at the same position in both probes but differ significantly in structure and hydrophobicity. The tandem VanS_B_ sensor protein bound to AF488-vancomycin with a *K*_D_ value of 11.0 μM, essentially identical to the affinity measured for BODIPY-FL-vancomycin, allowing us to rule out any nonspecific interactions between the sensor domain and the fluorophore.

Finally, we used isothermal titration calorimetry (ITC) to measure the binding of label-free vancomycin to the VanS_B_ sensor domain, obtaining *K*_D_ values of 20.7 μM for the single-copy sensor domain and 11.0 μM for the tandem sensor, in excellent agreement with the results of the fluorescence-anisotropy experiments (Fig. 5, *C* and *D*). Importantly, the related glycopeptide antibiotic teicoplanin does not bind the VanS_B_ sensor (Fig. S7), consistent with observation that teicoplanin does not induce resistance in type-B VRE (26). Taken together, these results clearly demonstrate a direct and specific binding interaction between vancomycin and the periplasmic sensor domain of VanS_B_.

## Discussion

Of the various organisms that comprise VRE, those displaying type-B resistance are perhaps the most rapidly growing threat. While at least 10 different VRE genotypes are known, infections in humans are predominantly caused by either type A or B, perhaps because both of these resistance phenotypes are associated with mobile genetic elements (56). Historically, type A has proven more prevalent than type B, but the incidence of the latter has been steadily rising (57, 58, 59, 60, 61). Type-B resistance confers high-level resistance against vancomycin, but not against related glycopeptide antibiotics (62). It is principally found in *Enterococcus faecium,* but has also been observed in other enterococcal species; in addition, nonenterococcal species, including gut anaerobes such as *Clostridioides* spp., can contain *vanB* genes, serving as reservoirs from which these genes can ultimately be transferred to enterococci (63).

The mechanisms by which any VRE evade vancomycin remain incompletely understood, with the largest open questions being how VanS recognizes the antibiotic and transduces this signal. Answers to these questions will likely inform the development of new therapeutic avenues. To date, most effort has been devoted to studying VanS proteins from type-A VRE and from nonenterococcal organisms, and these studies have not yet conclusively determined whether these proteins are activated by direct or indirect mechanisms. In contrast, the full-length VanS_B_ protein had not been well-characterized prior to this work. Here, we present definitive evidence for regulation of VanS_B_ activity *via* a direct interaction with vancomycin. Specifically, vancomycin increases VanS_B_’s autophosphorylation activity and slightly decreases its phosphatase activity; both effects will lead to increased levels of phospho-VanR_B_, thereby stimulating the transcription of the resistance operon. We further provide evidence of a direct physical interaction between vancomycin and VanS_B_. This is the first time that direct binding of the antibiotic has been linked to activity changes in a VanS protein.

Although we observed clear evidence for vancomycin binding to VanS_B_, the binding affinities and EC_50_ value for autokinase stimulation are higher than the ∼2 μM vancomycin concentration reported to induce transcription of resistance genes in type-B VRE (26). There are many potential explanations for this discrepancy. Different VanB isolates vary widely in their susceptibility to vancomycin (64), and it is possible that our chosen VanS_B_ sequence simply represents a relatively low-sensitivity vancomycin binder. It may also be true that our simplified *in-vitro* assay system, while useful, is not a perfect mimic of the environment found *in vivo*. For example, our assay system contains no peptidoglycan or lipid II; in bacterial cells, both these components would tend to recruit vancomycin to the vicinity of VanS_B_’s membrane-proximal sensor domain, giving rise to a high local concentration of the antibiotic. In particular, lipid II levels increase in response to vancomycin activity (65); in addition to recruiting vancomycin to the membrane surface, the lipid II could also potentially modulate VanS_B_ activity directly. Another possibility is that full activation may require the presence of both the antibiotic and certain peptidoglycan species, as is seen with the VanS protein from *Streptomyces coelicolor*, which is thought be activated by vancomycin in complex with a D-Ala-D-Ala-containing peptidoglycan precursor (66). Another factor to consider is that our NDs contain, on average, only two copies of the VanS_B_ molecule per disc, likely forming a homodimer; if a higher-order assembly of dimers is required for complete activation (35), this could not be achieved in our system. Alternatively, some other protein (encoded in either the *vanB* operon or the enterococcal genome) might be required to achieve maximum sensitivity. Finally, we note that our ND preparations use commercially available *E. coli* lipids, in which phosphatidylethanolamine is the predominant lipid species, along with smaller amounts of phosphatidylglycerol and cardiolipin (67). In contrast, membranes of enterococci feature primarily phosphatidylglycerol and cardiolipin (68), and this difference in lipid environment could conceivably affect VanS_B_ function.

Different VanS proteins perform equivalent functions in their respective VRE strains, but may do so by different mechanisms. The key to any mechanistic differences is likely to reside in the various proteins’ sensor domains. For example, while VanS_A_ and VanS_B_ share ∼19% sequence identity and 36% overall similarity, this similarity is most pronounced in the cytoplasmic regions of these proteins, whereas the periplasmic sensor domains are highly dissimilar in both size and sequence (Fig. S8). VanS_A_ is typical of most VanS proteins in that it contains a short periplasmic domain—in this case, ∼37 residues. In contrast, the VanS_B_ periplasmic domain is ∼101 residues in length. The VanS_B_ periplasmic region is predicted to adopt a PAS domain fold; this appears to be unique to type-B VanS proteins, as the periplasmic regions of VanS proteins from other VRE types are too short to accommodate such a structure (Supplementary Fig. S9). In fact, phylogenetic analysis reveals that VanS genes from type-B VRE form an outlier cluster, distinct from the VanS genes associated with other VRE types (69). VanS_B_ remains an outlier when compared with VanS proteins from organisms other than Enterococci, as they also lack large periplasmic domains (Supplementary Fig. S9). Interestingly, the VanS protein from one such organism, *S. coelicolor*, has been shown to interact directly with vancomycin *via* its periplasmic domain (70); however, this domain contains only approximately 27 residues, and thus is inconsistent with a VanS_B_-like structure. Hence, the PAS sensor domain that allows VanS_B_ to bind directly to vancomycin is evolutionarily distinct, making it difficult to extrapolate from the type-B example and draw mechanistic inferences about VanS proteins from other VRE types.

The elucidation of the VanS_B_ sensing mechanism has important translational implications. For example, the confirmation of a direct binding interaction between the antibiotic and the protein rationalizes why type-B VRE are only resistant to vancomycin, and not to other glycopeptide antibiotics that share vancomycin’s mechanism of action but differ in structure. This holds out the possibility of generating new “stealth antibiotics,” vancomycin derivatives that are not recognized by VanS_B_. Alternately, one can envision small molecules that block vancomycin binding to VanS_B_, acting as antibiotic adjuvants that restore vancomycin efficacy by suppressing VanRS signaling.

## Experimental procedures

### Expression constructs

Primers used for subcloning are shown in Table S1. pMSP1D1 was obtained from Addgene (catalog #20061). The genes for full-length VanS_B_ and VanR_B_ (Uniprot IDs Q47745 and Q47744, respectively) were codon-optimized for expression in *E. coli* cells by GenScript and subcloned into the in-house pETCH vector, producing expression constructs containing C-terminal His_6_ tags (71). To produce a tagless version of VanS_B_, the gene was inserted into the in-house pETHSUL vector, thereby fusing a cleavable His_6_-SUMO sequence to the N terminus of VanS_B_ (71). The cytosolic form of VanS_B_ (cVanS_B_) was previously determined to comprise residues M159-L447 (17), and the corresponding gene fragment was subcloned into pETCH. The periplasmic domain of VanS_B_ is located between the two transmembrane helices, which are predicted to consist of residues 10 to 28 and 135 to 155 (72). Therefore, to produce a tandem periplasmic-domain construct, two gene fragments were designed. Both encoded residues Q31 to G132 of VanS_B_ and also included an overlapping linker region encoding the linker sequence GSGGS connecting the C terminus of the first copy to the N terminus of the second copy. These fragments were assembled using the NEBuilder HiFi Assembly kit into pMal-c6T (NEB #N0378S). The resulting construct encodes a tobacco etch virus (TEV) protease-cleavable 6xHis-MBP tag fused to two copies of the VanS_B_ periplasmic domain. The single-copy construct was created by introducing the sequence TAAT *via* site-directed mutagenesis, placing an enhanced stop codon after the first copy of the VanS_B_ sensor domain (73). A similar strategy was used to produce tandem and single-copy constructs of the *B. subtilis* PhoR periplasmic domain (Uniprot P23545 aa 32–150), amplifying the appropriate region from *B. subtilis* subsp. *subtilis* strain 168 (American Type Culture Collection cat. no. 23857).

### Protein expression and purification

C terminally His-tagged VanS_B_ was purified following a protocol previously described for VanS_A_ (44), with minor modifications. Briefly, pelleted cells from 6-l cultures were resuspended in immobilized metal-ion affinity chromatography (IMAC)-A buffer (40 mM Tris pH 8, 300 mM NaCl, and 25 mM imidazole) containing EDTA-free protease inhibitor tablets (Thermo-Pierce) and then lysed in a C5 Emulsiflex cell homogenizer (Avestin, Inc.) at 10,000 to 15,000 psi. Membranes were isolated by centrifugation and homogenized in IMAC-A buffer, after which proteins were solubilized by the addition of 1% (w/v) DDM for 1 h at 4 °C. The solubilized membranes were centrifuged at 150K *g* for 50 min, and the resulting supernatant was syringe-filtered (0.45 μm) and loaded onto a 1-mL IMAC-HP column (Cytiva) equilibrated in IMAC-A buffer containing 0.2% DDM. Protein was eluted with a gradient from 25 to 350 mM imidazole.

To produce the tagless version of VanS_B,_ the pETHSUL-VanS_B_ plasmid was transformed into BL21(DE3) pLysS cells. Cells were grown in Terrific Broth media with 100 μg/ml ampicillin and 34 μg/ml chloramphenicol to an *A*_600_ of 0.8, after which the temperature was reduced to 16 °C. Once the *A*_600_ reached 1.2, 1 mM IPTG was added, and the cultures were shaken for 22 h, after which cells were harvested by centrifugation and frozen at −80 °C. After thawing, cells were lysed and the His_6_-SUMO-VanS_B_ protein was purified following the identical procedure used for the C terminally His-tagged VanS_B_ protein.

VanR_B_ was coexpressed with GroEL/S chaperones. pETCH-VanR_B_ and pGro7 (Takara Cat# 3340) were cotransformed into BL21(DE3) cells. Overnight cultures were diluted 1:500 in LB media containing 100 μg/ml ampicillin and 34 μg/ml chloramphenicol. Immediately after inoculation, arabinose (1 mg/ml) was added to induce chaperone expression. At *A*_600_ = 0.5, the temperature was lowered to 30 °C, IPTG was added (0.2 mM), and cells were incubated for 4 h. Cells harvested from 3-l cultures were resuspended in 40 mM Tris pH 8, 500 mM NaCl, 25 mM imidazole, 10% w/v glycerol, and 5 mM MgCl_2_ (Buffer IMAC-A2) containing 10 μg/ml DNase, 2 μg/ml RNase, and EDTA-free protease inhibitor tablets. The resuspended cells were lysed by homogenization, after which Triton-X100 was added to the lysate at a final concentration of 0.1% (v/v) and the sample incubated at 4 °C for 1 h. The lysate was centrifuged at 150K *g* for 1 h, filtered (0.45-μm), and loaded onto a 1-mL IMAC-HP column equilibrated in IMAC-A2 containing 0.1% Triton-X100. The column was washed with 20 CV of IMAC-A2 containing 0.1% Triton-X100, 20 CV of IMAC-A2 without detergent, and 20 CV of 10% IMAC-B2 (40 mM Tris pH 8, 500 mM NaCl, 350 mM imidazole, 10% glycerol, 5 mM MgCl_2_). VanR_B_ was eluted with a gradient from 10 to 100% IMAC-B2. Fractions containing VanR_B_ were pooled, concentrated to 5 ml, syringe-filtered (0.22-μm), and injected onto a Sephacryl S300 16/60 size-exclusion column (Cytiva) equilibrated in IMAC-A2 buffer lacking imidazole. Aliquots of VanR_B_ were flash-frozen in liquid nitrogen and stored at −80 °C.

All periplasmic-domain constructs were expressed in BL21(DE3) cells (Fig. S5*A*). Cells were grown in LB broth with 0.2% glucose at 37 °C to an *A*_600_ of ∼0.6, at which point temperature was reduced to 16 °C and IPTG was added to a final concentration of 0.2 mM. Cells were harvested after ∼22 h of shaking at 225 RPM. Cells from 2 l growths were lysed in buffer containing 20 mM Tris pH 7.5, 200 mM NaCl, 5 mM MgCl_2_, 1 mM EDTA, and protease inhibitors (Thermo Fisher Scientific #A32963). Cell lysate was clarified by centrifugation at 10,000*g* for 20 min then at 117,000*g* for 1 hour, after which the soluble supernatant was filtered (0.45 μm) and loaded onto a 42-mL column packed with amylose resin (NEB #E8021L) equilibrated with Buffer A3 (20 mM Tris pH 7.5, 100 mM NaCl). The column was washed with Buffer A3, then the captured protein was eluted in batch using Buffer A3 supplemented with 50 mM maltose. The eluted protein was mixed with 2 mg of TEV protease and dialyzed overnight against 1 l of IEX buffer (20 mM Tris pH 7.5) containing 1 mM DTT. The following day, the dialysate was loaded onto a 5-mL HiTrap Q HP column (Cytiva) equilibrated with IEX buffer. The column was first washed with IEX buffer, followed by IEX buffer supplemented with 100 mM NaCl. At this point, a 5-mL HiTrap IMAC HP column was attached downstream of the Q column and proteins were eluted using IEX buffer containing 600 mM NaCl. The IMAC column served to capture the TEV protease, the cleaved 6xHis-MBP tag, and any uncleaved protein during elution. The eluted protein was concentrated to 10 ml and loaded onto a Sephacryl-S100 26/60 column equilibrated with Buffer A3. Fractions containing the desired proteins were analyzed by SDS-PAGE for purity, pooled, and concentrated to at least 5 mg/ml.

The cytosolic domain of VanS_B_ (cVanS_B_) was purified similarly to VanR_B_ with one modification: 21 mM *N*-decyl-*N*,*N*-dimethylamine-*N*-oxide (DDAO) was added to the lysate and buffer IMAC-A2. MSP1D1 was expressed and purified as previously described (32, 33). The cytosolic domain of VanS_A_ was purified as described (44).

### Nanodisc assembly and purification

Four ml of a 25 mg/ml chloroform solution of *E. coli* total lipid extract (Avanti Polar Lipids, Inc.) were placed in a clean glass vial. The chloroform was evaporated with a stream of N_2_ gas and the vial placed in a lyophilizer overnight. The dried lipids were resuspended in 3.75 ml of 4% (w/v) DDM in ultrapure water. The suspension was sonicated for 10 min, freeze-thawed, sonicated for an additional 10 min, and then stored on ice. Lipid concentration was determined by phosphorous assay (74). NDs were assembled by combining VanS, MSP1D1, and *E. coli* lipids in a mole ratio of 1:10:600 in a final volume of 500 μl, with a final VanS concentration of 4 μM. After a 40-min incubation at room temperature, the 500-μl ND mixture was added to 200 μl wet Bio-Beads (Bio-Rad Cat# 1523920) in a fresh 1.5-mL tube. Bio-Beads were prepared as described (75). The Bio-Bead-containing mixtures were rotated end-over-end at 4 °C overnight. The following day, Bio-Beads were removed by filtration and the concentration of imidazole was reduced to 30 mM by diluting the sample with cold ND buffer (20 mM Tris pH 7.4, 150 mM NaCl). The sample was syringe-filtered (0.22 μm) and applied to a gravity-flow column containing a 600-μl bed volume of nickel-charged resin equilibrated in IMAC A buffer. The column was washed with 4 ml of cold IMAC A and the VanS-containing NDs were eluted with 1.8 ml of cold 1:1 mixture of IMAC A and IMAC B buffers. The eluate was concentrated to 250 μl using a 100-kDa MWCO 0.5-mL concentrator, filtered (0.22 μm), and injected onto a Superdex 200 10/300 column (Cytiva) equilibrated in ND buffer at room temperature. Peak fractions were pooled, concentrated, quantified, and used for activity assays.

To generate NDs containing untagged VanS_B_, assemblies were performed as previously described with the following modifications. A mole ratio of 1:10:700 was used in a final volume of 500 μl, with a His_6_-SUMO-VanS_B_ concentration of 4 μM. The 500-μl ND mixture was added to 300 μl of wet BioBeads. After syringe-filtration (0.22 μm), the sample was applied to a 1-mL IMAC HiTrap HP column equilibrated in IMAC A Buffer. The column was washed with cold IMAC A and the His_6_-SUMO-VanS_B_-containing NDs were eluted with cold IMAC B buffer. The eluate was dialyzed against 2 l of 20 mM Tris pH 7.4, 150 mM NaCl overnight at 4 °C in a 3K-MWCO dialysis cassette (Thermo Slide-A-Lyzer). Prior to dialysis, 1 mg of purified SUMO protease (dtUD1; 71) was added to cleave the His_6_-SUMO tag. The dialysate was then applied to the same IMAC column and the flowthrough was collected. The sample was concentrated to 250 μl using a 100-kDa MWCO 0.5-mL concentrator, filtered (0.22 μm), and injected onto a Superdex 200 10/300 column as described above.

### Autophosphorylation assays

The autophosphorylation assays using ATP*γ*S were performed as described (44), using the following stock solutions: 5X reaction buffer (250 mM KCl, 50 mM MgCl_2_, 250 mM Tris pH = 7.4), 10 mM ATPγS (Abcam ab138911) in ultrapure water, 1 mM vancomycin in ND buffer (20 mM Tris pH 7.4, 150 mM NaCl), 0.5 M EDTA pH 8, and 50 mM p-nitrobenzyl mesylate (PNBM; Abcam ab138910) in 100% dimethyl sulfoxide (DMSO).

To determine the effect of vancomycin on autophosphorylation activity, two separate master-mix reactions were created, of 66 μl each. Each reaction contained 1x reaction buffer, ATPγS at a final concentration of 1 mM, and VanS-containing NDs at a final concentration of 0.3 to 0.5 μM. One reaction contained vancomycin at a final concentration of 100 μM and the other contained an equivalent volume of ND buffer to ensure the salt concentration was consistent. At each time point, 15 μl was transferred from the reaction to a designated tube that contained 3 μl of 0.5 M EDTA pH 8 to quench the reaction. After all of the time points had been taken, 2 μl of PNBM was added to each tube and the mixture was incubated at room temperature for 1 h. The samples were loaded onto a Bio-Rad 12% precast gel and run at 200 V with cold running buffer at 4 °C, and subsequently transferred onto polyvinylidene fluoride (PVDF) membrane for 1 h at 100 V. The membrane was rocked in blocking buffer (5% milk in Tris-buffered saline, 0.1% Tween 20 (TBST)) for 1 h, rocked in anti-PNBM (Abcam ab92570) at 1:5000 in 1% milk-TBST for 1 h, washed in TBST 6 × 8 min, rocked in anti-GAR (Jackson ImmunoResearch horseradish peroxidase (HRP)-GAR IgG 111–035–003) at 1:1000 in 1% milk-TBST for 1 h, and washed in TBST 6 × 8 min. The membranes were rocked in enhanced chemiluminescence detection reagent for 1 min prior to exposing the membranes using a Syngene imager. Blots were stripped and probed with HRP-conjugated anti-His antibody at 1:20,000 dilution in 1% milk TBST. The band intensities and background intensities were quantified by ImageJ and the band intensities for each time point were background-corrected. To determine the fold-stimulation by vancomycin, bands representing autophosphorylation in the presence of vancomycin were normalized to corresponding time points representing autophosphorylation in the absence of vancomycin.

A slight variation to the above protocol was introduced in order to determine the EC_50_ of vancomycin. Multiple individual 15-μl autophosphorylation reactions were initiated in the presence of varying concentrations of vancomycin. The start of the reactions was staggered by 30 s, and each reaction was allowed to proceed for 30 min, at which point 3 μl of EDTA was added to quench.

Autophosphorylation experiments using *γ*-^32^P-labeled ATP (PerkinElmer BLU002Z250UC, 10 mCi/ml) were set up similarly to those using ATP*γ*S. A 50-μl autophosphorylation reaction contained 1X reaction buffer, 2 μl of the ^32^P-ATP stock, 1 mM cold ATP, and VanS_B_-containing NDs at 0.5 μM. Reactions were quenched by the simultaneous addition of EDTA and SDS-PAGE loading buffer and the samples analyzed by SDS-PAGE and autoradiography.

### Dephosphorylation assays

The VanR_B_ protein as purified from *E. coli* was observed to be partially phosphorylated, presumably as a result of nonspecific phosphorylation by either endogenous histidine kinases or nonenzymatic phosphoryl donors (Fig. S10). This VanR_B_ preparation was therefore used to assess the dephosphorylation activity of VanS_B_. Partially phosphorylated VanR_B_ protein was incubated at room temperature with VanS_B_, in reactions consisting of 1x reaction buffer, VanS_B_-containing NDs at 0.5 μM, and VanR_B_ at 1 μM. Reactions were quenched at various time points by the addition of SDS-PAGE loading buffer, after which samples were analyzed by electrophoresis on Phos-tag gels (Fujifilm Cat. # 195–17991). Gels were run in cold, fresh running buffer at 200 V for approximately 2 to 2.5 h at 4 °C, and then stained with Coomassie brilliant blue.

### Phosphotransfer assays

Purified VanS_B_ was reconstituted into NDs and an autophosphorylation assay was prepared as follows: 25 μl of γ^32^P-ATP (1.7 μM, 10 mCi/ml) was mixed with 6.25 μl of 50 mM cold ATP to give a 10 mM ATP stock solution. Fifteen microliters of this stock solution was combined with VanS_B_ in 1x reaction buffer to give a total volume of 150 μl and a final VanS_B_ concentration of 10 μM. The reaction mixture was incubated at room temperature for 1 h, after which excess nucleotide was removed by two passes through 0.5-mL Zeba Spin desalting columns (Thermo Fisher Scientific) equilibrated in 1x reaction buffer. For each phosphotransfer experiment, an aliquot of this phospho-VanS_B_ was mixed with vancomycin or buffer, at which point a zero time point was removed. The reaction was then initiated by addition of VanR_B_, with the final concentrations in the reaction mixture being 1.5 μM phospho-VanS_B_ and 3 μM VanR_B_. Briefly, 10-μl time points were removed and mixed with 3 μl of 0.5 M EDTA pH 8 to quench the reaction. Samples were analyzed by SDS-PAGE, using 12% Bis-Tris gels (GenScript SurePAGE). The gels were dried and analyzed by autoradiography.

### Liquid-chromatography mass spectrometry

Molecules were analyzed on a Waters Acquity I-Class UPLC system coupled to a SYNAPT G2-Si HDMS mass spectrometer in positive ion mode with a heated electrospray ionization source in a Z-spray configuration. For proteins, LC separation was performed on a Waters Acquity UPLC Protein BEH C_4_ 1.7 μm 2.1 x 50 mm column maintained at 40 °C; a 0.2 ml/min gradient of 80/20 to 30/70 A/B in 20 min was used, followed by washing and reconditioning the column. For vancomycin, LC separation was performed on a Waters Acquity UPLC BEH C_18_ 1.7 μm 2.1 x 50 mm column using an 0.6 ml/min gradient of 95/5 to 15/85 A/B over the course of 4 min. Eluent A is 0.1% v/v formic acid in water and B is 0.1% v/v formic acid in acetonitrile. Conditions on the mass spectrometer were as follows: capillary voltage 0.5 kV, sampling cone 40 V, source offset 80 V, source 120 °C, desolvation 250 °C, cone gas 0 l/h, desolvation gas 1000 l/h, and nebulizer 6.5 bar. The analyzer was operated in resolution mode and low energy data were collected between 100 and 2000 Da at 0.2 s scan time. For vancomycin, MS^e^ data were collected using a 20-40V ramp trap collision energy. Masses were extracted from the TOF MS TICs using a 0.005 Da abs width. Protein electrospray ionization data were deconvoluted using MaxEnt1 in Masslynx 4.1 (Waters Corporation).

### Photolabeling reactions

Photoprobes containing diazirine groups attached to either the amino group of vancomycin’s vancosamine sugar (Probe V) or to the antibiotic’s N terminus (Probe N) were prepared as described (51). Photolabeling was initiated by irradiation with 365-nm light, and samples were analyzed by mass spectrometry and Western blotting. 50-μl reactions containing 20 μM protein and 50 μM photoprobe were prepared in 20 mM Tris pH 7.5, 100 mM NaCl. The full-length VanS_B_ protein was reconstituted into NDs prior to photolabeling and used at a concentration of 20 μM. The reactions were placed into glass depression spot plates, which were placed atop a heavy aluminum plate packed in ice, to keep reactions cold and to limit evaporation. Samples were irradiated at 365 nm using a Stratagene Stratalinker 2400 UV Crosslinker for the times specified. For competition experiments with vancomycin, samples were irradiated for 2 min. All samples were diluted 1:50 for subsequent analysis by mass spectrometry and Western blotting.

For Western blot analysis, 100 ng of protein were loaded onto a 12% SDS-PAGE gel and electrophoresed at 180 V. While the gel was running, a 0.2 μm PVDF membrane (Cytiva #10600021) was prepared by soaking in 100% methanol for 1 min then in water for 2 min. The membrane was then equilibrated in transfer buffer for 15 min (25 mM Tris pH 8.3, 192 mM glycine, and 15% methanol). Proteins were transferred from the gel to the membrane for 1 hour at 100 V, after which the membrane was rocked in blocking buffer (5% milk in 20 mM Tris pH 7.6, 150 mM NaCl, 0.1% Tween-20 (TBST)) for 30 min. The membrane was then rocked overnight at 4 °C in a solution containing a sheep anti-vancomycin polyclonal antibody (Bio-Rad # 9520–0004) diluted 1:1000 in blocking buffer. The following day, the membrane was washed in TBST 3 × 10 min and then rocked at room temperature for 1 hour in solution containing a rabbit anti-sheep HRP-conjugated secondary antibody (Invitrogen # 31480), diluted 1:5000 in blocking buffer. The membrane was then washed in TBST 3 × 10 min and placed in peroxidase substrate solution (Pierce #32209) for 1 min before chemiluminescent detection.

### Vancomycin binding assays

Fluorescence anisotropy experiments were conducted in 20-μl volumes containing 100 nM BODIPY-FL-vancomycin (Thermo Fisher Scientific, #V34850) or Alexa Fluor 488-vancomycin in 20 mM Tris pH 7.5 buffer containing 100 mM NaCl and 0.01% Triton X-100, using black 384-well small-volume plates (Greiner Bio-One, # 784077). Anisotropy measurements were conducted at room temperature using a Tecan Spark microplate reader. Samples were excited at 490 nm and emission read at 525 nm, using a 10-nm bandpass for both emission and excitation. A 510-nm dichroic mirror was used to condition the emitted signal. Single-copy and tandem-copy versions of the VanS_B_ periplasmic domain were used with the BODIPY-FL probe; the tandem-copy protein was used with the AlexaFluor 488 probe to confirm that there was no dependence of binding on the specific identity of the fluorophore.

ITC experiments were carried out using a MicroCal VP-ITC calorimeter (Malvern Panalytical). Protein and vancomycin stock solutions were exhaustively dialyzed against 20 mM Bicine pH 7.5. The sample cell was filled with 200 μM vancomycin, and the injection syringe was loaded with 2 mM VanS_B_ single-copy periplasmic-domain protein or 1 mM VanS_B_ tandem periplasmic-domain protein. An initial 5-μl injection was followed by twenty 10-μl injections at 15 °C, with 4 min between each injection and spinning at 340 RPM. Binding signatures were analyzed using the MicroCal Analysis software and the data were fit using a single-site model. Estimates of binding stoichiometry (N values) varied during the fitting, depending on the experimental run and the fitting protocols used, with final values suggesting stoichiometries below 1:1. This behavior likely reflects the propensity of multivariable fitting procedures to accommodate noise or error in some parameters by adjusting other parameters; it may also indicate that the periplasmic-domain constructs are not highly stable over the time course of the calorimetry experiment.

### Modeling of the VanS_B_ periplasmic sensor domain

The sequence of the VanS_B_ periplasmic domain (residues 31–132, Uniprot Q47745) was used for protein threading (template-based) experiments, using I-TASSER, RaptorX, Phyre2, and SparksX (76, 77, 78, 79). All the threading approaches used gave predicted structures with similar folds; selected examples are shown in Figure 5B. One of the templates chosen most frequently by the different threading methods was the crystal structure of the periplasmic sensor domain of PhoR from *B. subtilis* (Protein Data Bank ID 3CWF), which prompted our use of this protein as a negative control that has a similar fold but a different function. For *ab initio* (template-independent) modeling studies, we used Quark, RoseTTAFold, and AlphaFold2 (80, 81, 82). In particular, AlphaFold2 and RoseTTAFold produced very similar structures, with an RMSD for all C*α* positions of 1.63 Å. Given that sensor histidine kinases are widely understood to be obligate dimers, we used AlphaFold2 to generate a model for the dimer, which is shown in Figure 5C.

### Preparation of AF488-labeled vancomycin

Vancomycin was dissolved at a concentration of 25 mg/ml in 90 mM sodium phosphate, pH 8. One mg of AF-488 NHS ester (BroadPharm, cat. no. BP-24307) was dissolved in 50 μl of DMSO. Briefly, 150 μl of the vancomycin solution (3.75 mg, 2.6 μmol) was added to the AF-488 NHS ester (1 mg, 1.6 μmol) and incubated at room temperature, protected from light, for 2 h. The reaction mixture was then frozen. After thawing, it was diluted 1:10 with water, after which DMSO was added to a final concentration of 20% (v/v). The material was then purified by reverse-phase chromatography on a 1 × 20 cm Ultrasphere 5-μm ODS column (Hichrom), using a gradient from 5% A to 100% B (solvent A = 0.25% formic acid in water, solvent B = 0.25% formic acid in acetonitrile). Two peaks having identical masses were isolated, corresponding to the addition of the fluorophore to either the vancosamine sugar or the antibiotic N-terminus ([M+2H]^2+^ calculated, 983.3588; observed, 983.6919). To distinguish these two species, the sugars were removed from the aglycon macrocycle by acid hydrolysis, after which the mass of the aglycon was determined by mass spectrometry (see Fig. S6; 51, 83). We were able to cleanly isolate the species having the fluorophore attached to the vancosamine sugar (∼95% pure); however, we were unable to isolate clean fractions containing the species labeled at the antibiotic’s N-terminus, and therefore did not use this species for binding experiments.

## Data availability

All data are available in the main text or the supplementary materials.

## Supporting information

This article contains supporting information.

## Conflict of interest

The authors declare that they have no conflicts of interest with the contents of this article.
