# Supplementary material for: The VanS sensor histidine kinase from type-B vancomycin-resistant enterococci recognizes vancomycin directly
Source: J Biol Chem. 2025 May 22;301(6):110276. doi: 10.1016/j.jbc.2025.110276 (PMC12212144; doi:10.1016/j.jbc.2025.110276)
Supplement: Supplementary Figs S1–S10 and Table S1 [file mmc1.pdf]

Supporting Information

The VanS sensor histidine kinase from type-B vancomycin-resistant enterococci recognizes vancomycin directly

Lina J. Maciunas, Photis Rotsides, Elizabeth J. D'Lauro, Samantha Brady, Joris Beld, and Patrick J. Loll*

*Corresponding author: Patrick J. Loll, pjl28@drexel.edu

This PDF file includes:

Fig. S1. *Autokinase-deficient mutants of* $VanS_B$ S2
Fig. S2. *Phosphotransfer from* $VanS_B$ *to* $VanR_B$ S3
Fig. S3. *Vancomycin stimulation of* $VanS_B$ *activity:* ^{32}*P-ATP assay* S4
Fig. S4. *A* $VanS_B$ *construct lacking a His-tag behaves similarly to tagged protein* S5
Fig. S5. $VanS_B$ *periplasmic domain modeling and purification* S6
Fig. S6. *Novel AF488-based fluorescence anisotropy probe* S7
Fig. S7. *ITC control experiments* S8
Fig. S8. *Pairwise sequence alignment of* $VanS_A$ *and* $VanS_B$ S9
Fig. S9. *Lengths of the sensor domains in different VanS proteins* S10
Fig. S10. $VanR_B$ *is partially phosphorylated in E. coli* S11
Table S1. *Primers used to prepare expression constructs.* S12

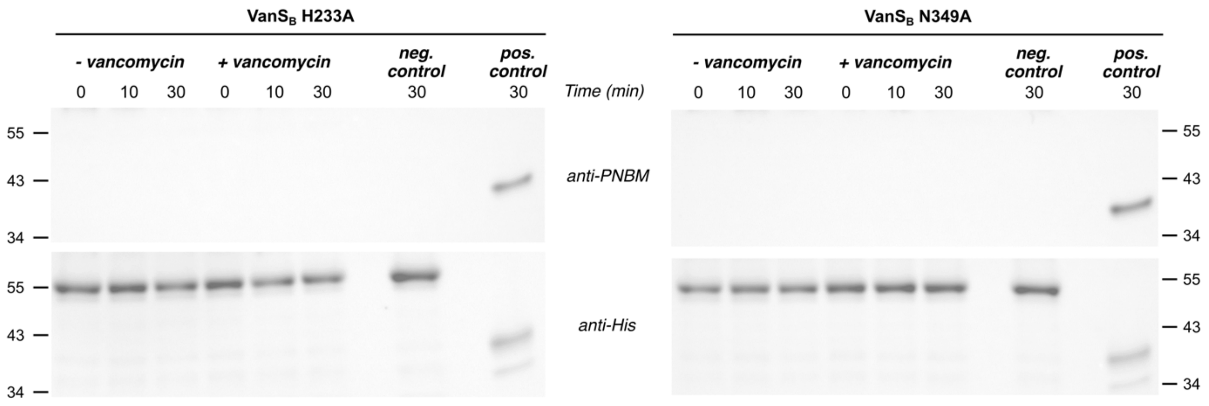

Figure S1. Autokinase-deficient mutants of VanS$_B$. Autophosphorylation assays were carried out for the H233A and N349A mutants of VanS$_B$ reconstituted into nanodiscs, using the ATPγS assay as described in the Methods section. Assays were performed in triplicate; representative blots are shown. The equivalent experiments for the wild-type enzyme can be seen in Figure 3A. For these experiments, the negative controls consisted of the purified, reconstituted VanS$_B$ enzyme with no ATPγS; the positive control used the purified soluble cytosolic domain of VanS$_A$ at a concentration of 0.5 μM.

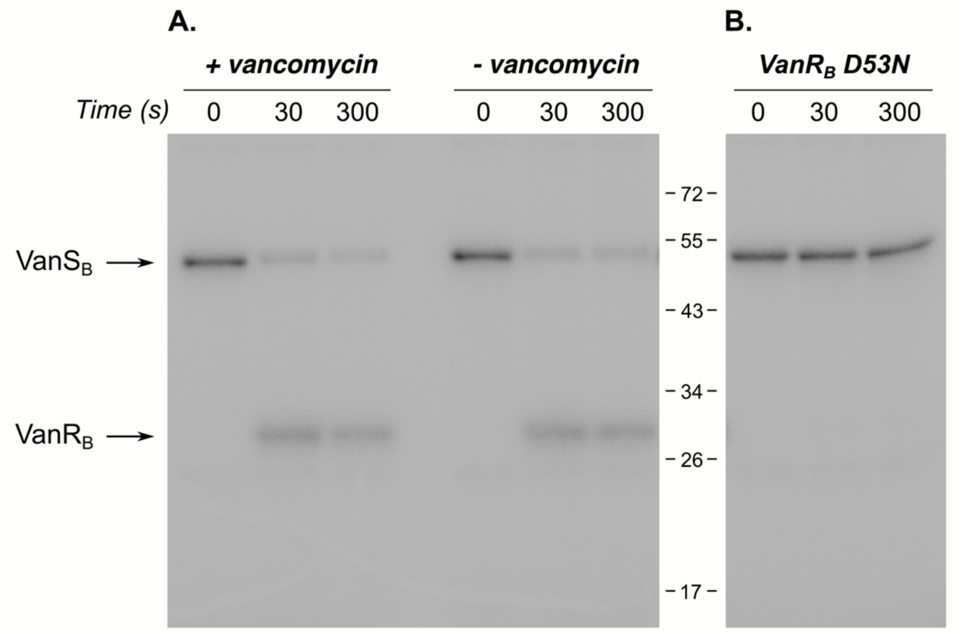

Figure S2. Phosphotransfer from VanS$_B$ to VanR$_B$. Purified VanS$_B$ in nanodiscs was allowed to autophosphorylate in the presence of [γ-^{32}P] ATP, after which unreacted nucleotide was removed. The phospho-VanS$_B$ was mixed with purified VanR$_B$ and samples were removed and quenched at the times indicated, and analyzed by SDS-PAGE and autoradiography. (A) Phosphotransfer to wild-type VanR$_B$ was analyzed in the presence or absence of 100 µM vancomycin. (B) Phosphotransfer to the D53N mutant of VanR$_B$ in the absence of vancomycin.

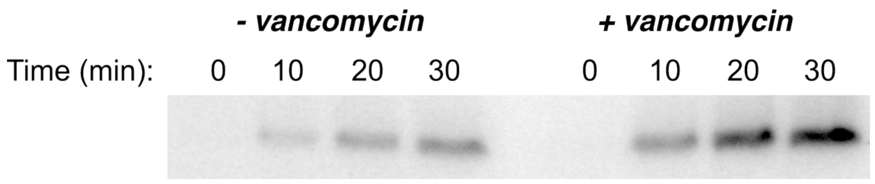

Figure S3. Stimulation of VanS$_B$ autophosphorylation activity by vancomycin: ^{32}P-ATP assay. VanS$_B$ in nanodiscs was incubated with a mixture of ^{32}P-labeled ATP and cold ATP, ± 100 μM vancomycin, for 0, 10, 20, and 30 minutes. The reactions were subjected to SDS-PAGE and dried onto a nitrocellulose membrane, and the membrane was exposed overnight. The stimulatory effect of vancomycin on the autophosphorylation activity of VanS$_B$ is still observed when the native substrate is used.

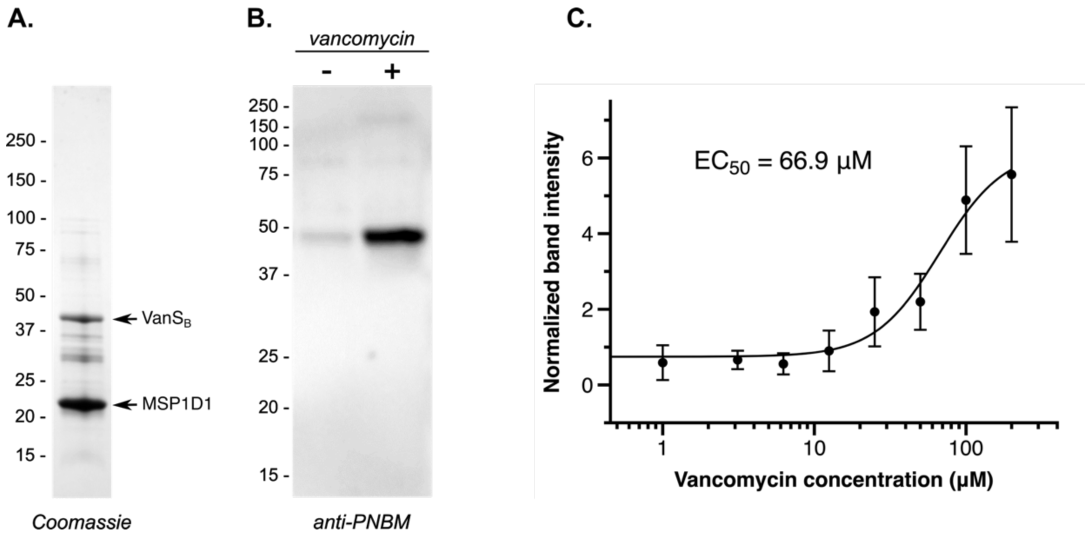

Figure S4. Full-length VanS$_B$ lacking a C-terminal His$_6$ tag behaves comparably to the tagged enzyme. (*A*) Coomassie-stained gel showing the nanodisc preparation for the tagless VanS$_B$ protein. (*B*) Representative Western blot showing the autophosphorylation activity of the tagless VanS$_B$ nanodisc preparation ± 100 μM vancomycin. (*C*) Dose-response curve showing the dependence of VanS$_B$ autophosphorylation upon vancomycin; error bars correspond to standard deviations reflecting two independent triplicate measurements. Vancomycin stimulates the activity of the tagless VanS$_B$ protein to a similar degree as that seen with the C-terminally His-tagged protein.

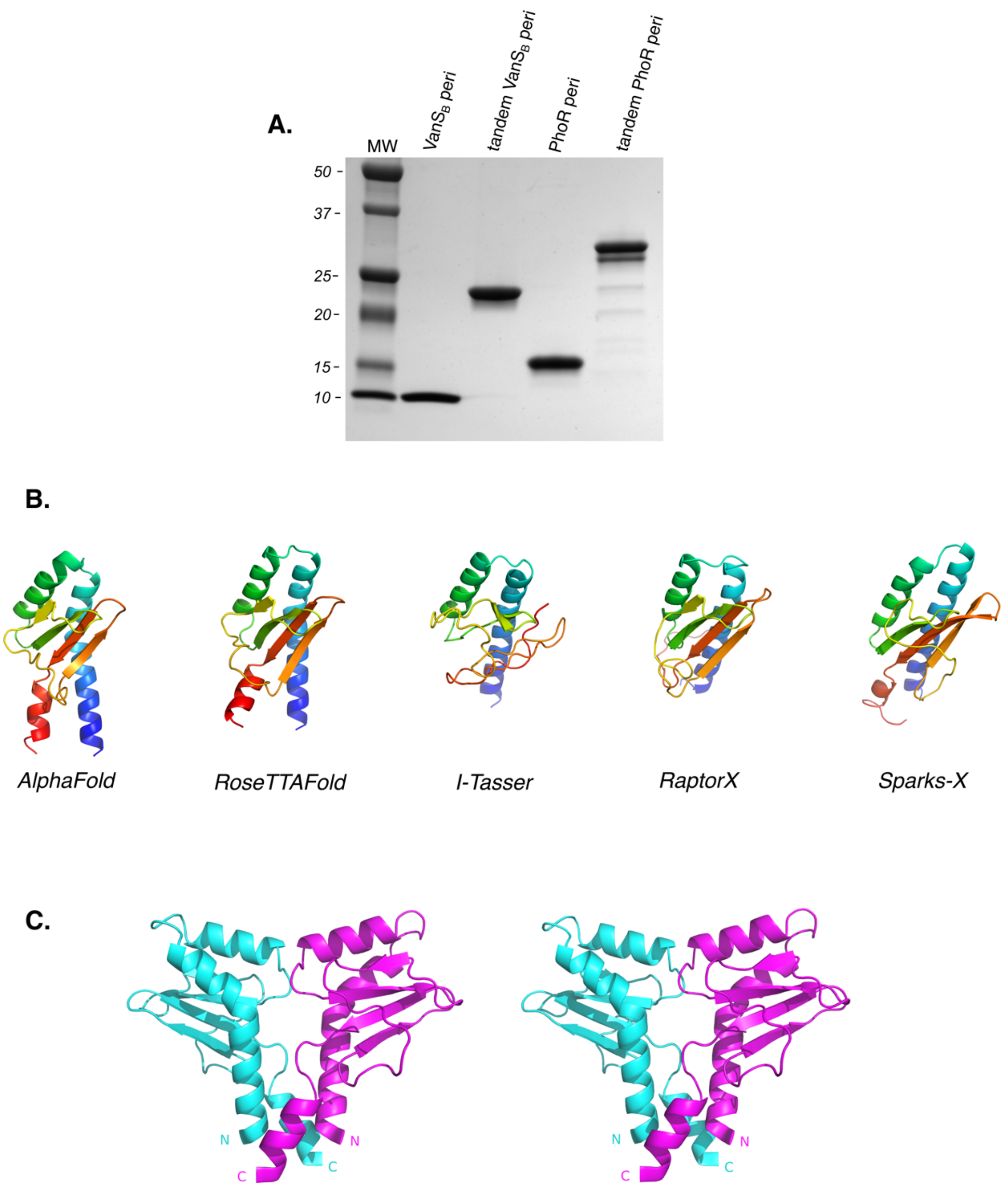

Figure S5. The VanS_B periplasmic sensor domain. A) Purified periplasmic sensor-domain constructs. Shown is a Coomassie-stained SDS PAGE gel; each lane contains 5 µg of the purified periplasmic-domain construct indicated. Molecular-weight markers are shown at left. B) Models of the VanS_B periiplasmic domain produced by selected programs. Each model is colored using a rainbow scheme, in which the color gradually changes from blue at the N-terminus to red at the C-terminus. C) Divergent stereo view of the AlphaFold model of the VanS_B periplasmic domain dimer. Shown are residues 35-135, with the two protomers being colored magenta and cyan. Positions of the N- and C-termini are indicated.

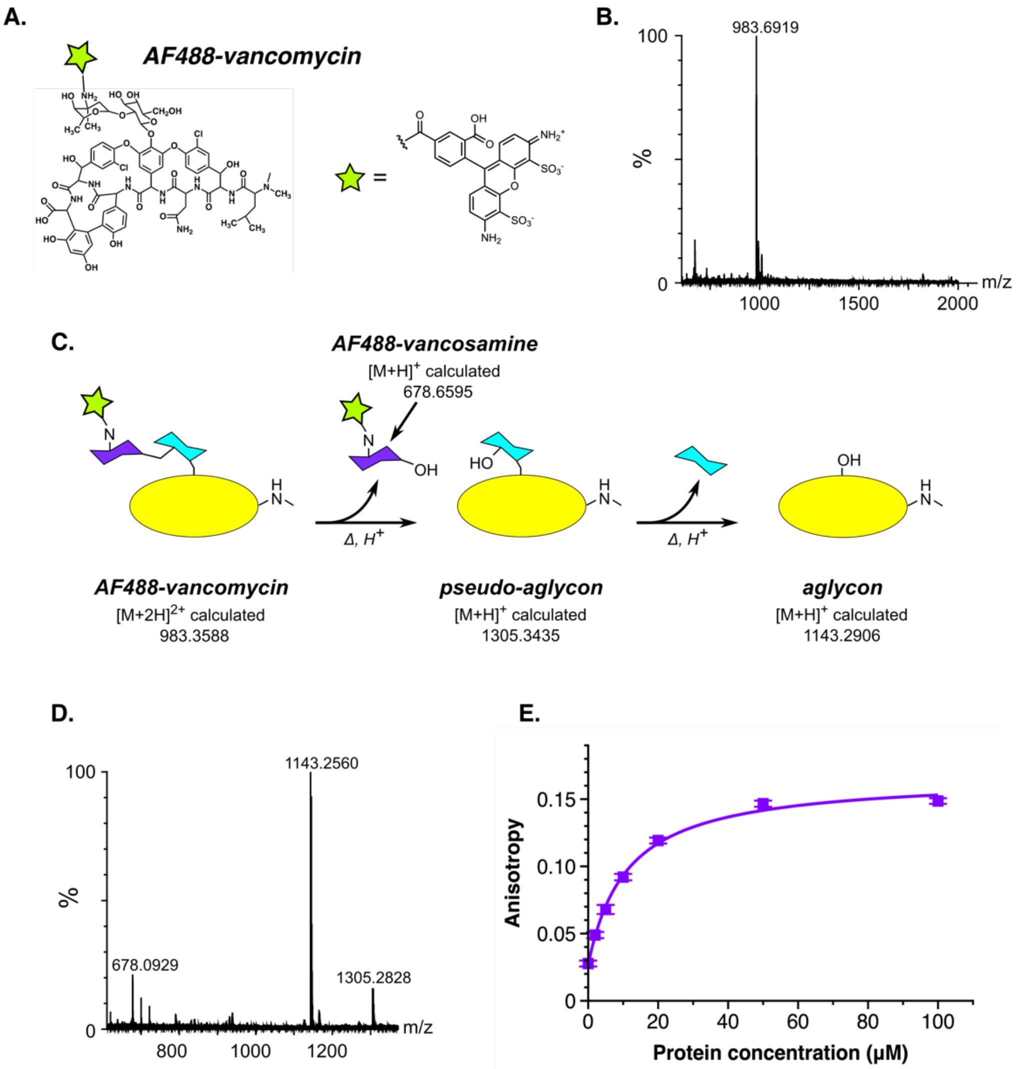

Figure S6. A new AF488-based fluorescence-anisotropy probe. A) Structure of the new AF488-vancomycin fluorescence-anisotropy probe. B) Confirmation of mass of the probe (calculated mass, 983.3588; observed, 983.6919). C) Scheme for determining the site of attachment of the AF488 fluorophore. There are two amines in vancomycin that can react with the NHS-AF488: A primary amine in the vancosamine sugar and a secondary amine at the N-terminus of the peptide. Acid hydrolysis will release the sugars, making it possible to use mass spectrometry to determine whether the dye is attached to the sugar or the aglycon. D) Mass spectrum of the acid hydrolysis products, showing that the dye is attached to the vancosamine sugar. E) Binding of the tandem $VanS_B$ sensor domain to AF488-containing probe, as measuring by change in fluorescence anisotropy. Fit to a single binding-site model is shown; estimated K_D value is 11.0 μM.

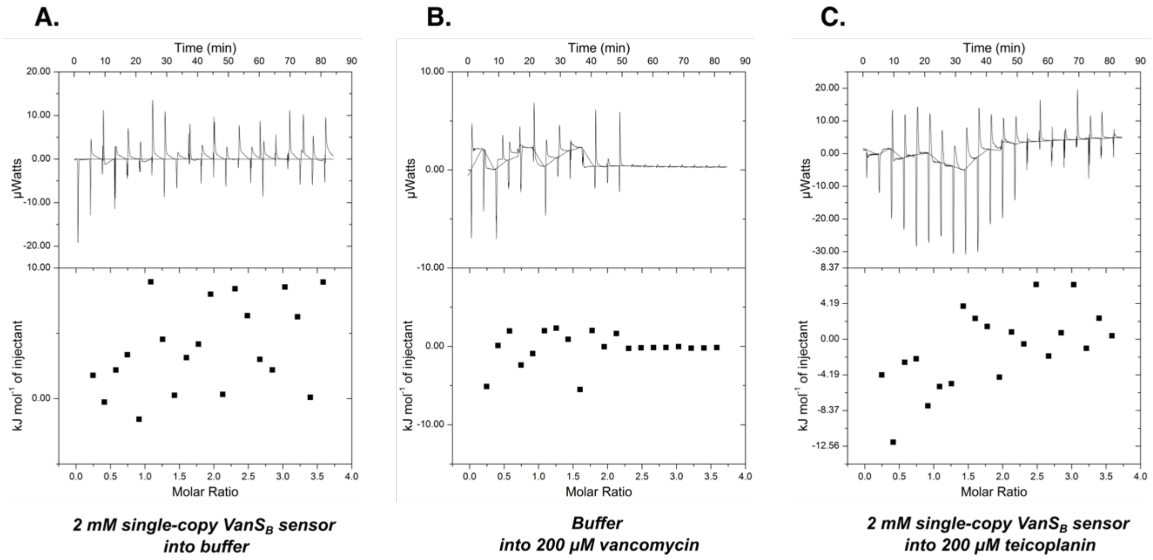

Figure S7. ITC control experiments. (A) Injection of 2 mM $VanS_B$ sensor domain into 20 mM Bicine, pH 7.5. (B) Injection of 20 mM Bicine, pH 7.5 into 200 µM vancomycin. (C) Injection of 2 mM $VanS_B$ sensor domain into 200 µM teicoplanin.

```
VanSA    1 MVIKLKNKKNDYSKLERK-------LYMYIVAIVVVAIV-------FVLYIRSMIRG---KLGDWILSILENKYDLNHLDAMKLYQYSIR----------
                         :|||        |  || :::| :        || | |:|      |    :: :::|   |:  :   |: |
VanSB    1 --------------MERKGIFIKVFSYTIIVLLLLVGVTATLFAQQFVSYFRAMEAQQTVKSYQPLVELIQNSDRLDMQEVAGLFHYNNQSFEFYIEDKE
                                  <------ TM1 ------>

VanSA   71 ----------------------------------------------NNIDIFIYVAIVISILILCRVMLSKFAKYFDEINTGIDVLIQNEDKQ-------
                                                           |   : :||::   :||  : ::      :: | |  |  : :|
VanSB   87 GSVLYATPNADTSNSVRPDFLYVVHRDDNISIVAQSKAGVGLLYQGLTIRGIVMIAIMVVFSLLCAYIFAR------QMTTPIKALADSANKMANLKEVP
                                                       <------- TM2 ------->

                                                                ★
VanSA  121 --IELSAEMDVMEQKLNT----LKRTLEKREQDAKLAEQRKNDVVMYL----AHDIKTPLTSIIGYL-SLLDEAPDMPVDQKAKYVHITLDKAYRLEQLI
             :|   |:  :   :::    || |: : | |    |    :   |     :|::|||: ::   |  :|:   |      :||:   :   |  : |
VanSB  181 PPLERKDELGALAHDMHSMYIRLKETIARLE-DEIAREHELEETQRYFFAAASHELKTPIAAVSVLLEGMLENIGD--YKDHSKYLRECIKMMDRQGKTI
                                                                ★

VanSA  210 DEFFEITRYNLQTITLTKTHIDLYYMLVQMTDEFYPQLSAHGKQAVIHAPEDLTVSGDPDKLARVFNNILKNAAAYSEDNSIIDITAGLSGDVVSIEFKN
            |  |:   |   |      :|:   : ::  :|     |: :: |   |    |  ||  : :  :|:: ||   :     :|  :    :   :   |
VanSB  278 SEILELVSLNDGRIVPIAEPLDIGRTVAELLPDFQTLAEANNQRFVTDIPAGQIVLSDPKLIQKALSNVILNAVQNTPQGGEVRIWSEPGAEKYRLSVLN

VanSA  310 TG-SIPKDKLAAIFEKFYRLDNARSSDTGGAGLGLAIAKEIIVQHGGQIYAESNDNYTTFRVELPAMPDLVDKRRS    384
            |  |    |: :|  |||:| |||  :| :|||||| :: :     |   |:  :   | ::||    |
VanSB  378 MGVHIDDTALSKLFIPFYRIDQARSRKSGRSGLGLAIVQKTLDAMSLQYALENTSDGVLFWLDLPPTSTL------    447
```

Figure S8. Pairwise sequence alignment of VanS$_A$ and VanS$_B$. Alignment was calculated using EMBOSS-Needle. Vertical lines mark identities, while double dots show residues of similar character. The predicted positions of the two transmembrane helices of VanS$_B$ are highlighted in red; the periplasmic sensor domain lies between these two helices. The histidine that is the target of autophosphorylation is indicated with a star.

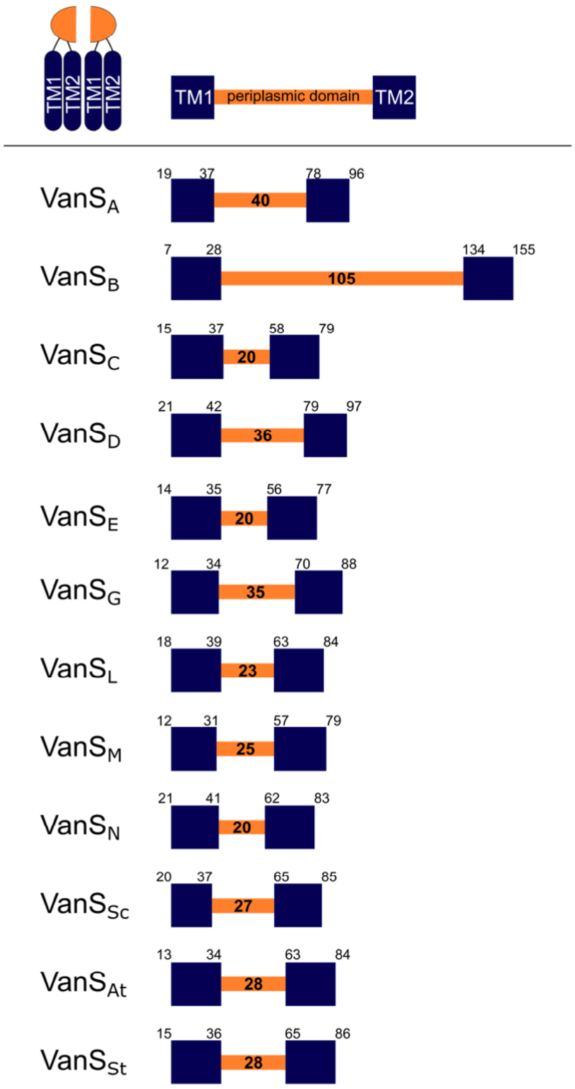

Figure S9. Lengths of the sensor domains in different VanS proteins. (*Top*) Cartoon representing the generic structure of the sensing domain of a VanS protein, in which the periplasmic region is located between transmembrane helix 1 (TM1) and transmembrane helix 2 (TM2). Two such domains are expected to associated in a functional dimer. (*Bottom*) Schematic representations for the sensor domains of individual VanS proteins. The positions of the transmembrane helices were determined using CCTOP [2], and are noted in each cartoon. The lengths of different VanS periplasmic sensor domains vary in length from 20-105 amino acids; the number of residues in each is indicated in bold. Accession numbers for the sequences used: VanS$_A$, Q06240.1; VanS$_B$, Q47745.1; VanS$_C$, AAF86642.1; VanS$_D$, AAD42181.1; VanS$_E$, AAL27446.1; VanS$_G$, AAQ16269.1; VanS$_L$, ABX54692.1; VanS$_M$, ACL82958.1; VanS$_N$, AEP40504.1. Sc, At, and St refer to the VanS proteins from *Steptomyces coelicolor*, *Actinoplanes teichomyceticus*, and *Streptomyces toyocaensis*, accession numbers TYP17068, TWG11274, and AAM80542, respectively.

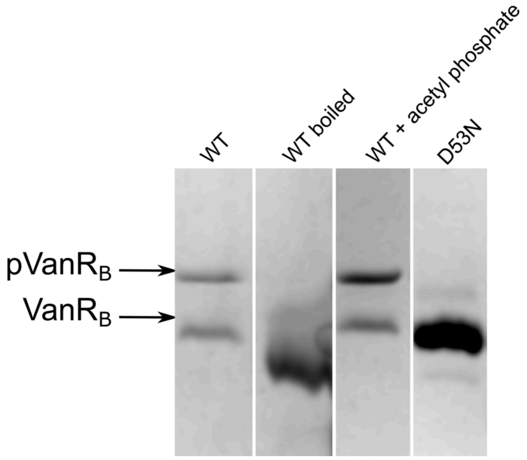

Figure S10. VanR$_B$ is partially phosphorylated in E. coli. Coomassie-stained Phos-tag^{TM} gel showing that VanR$_B$ as isolated from *E. coli* is partially phosphorylated. From left to right: 1) Wild-type (WT) VanR$_B$ runs as two distinct bands; 2) Boiling WT VanR$_B$ converts the two bands to a single species. Boiling is expected to remove the labile phosphoryl group; 3) WT VanR$_B$ was phosphorylated with the small-molecule phosphoryl donor acetyl phosphate, which increases the intensity of the upper band; 4) The nonphosphorylatable VanR$_B$ D53N mutant exhibits no upper band, consistent with that band corresponding to phospho-VanR$_B$.

Table S1. Primers used to prepare expression constructs.

Primer	Primer name	Primer Sequence
**1**	VanR$_B$_pETCH_F	5′-TTTTTTTTCCATGGCGATACGAATTCTACTTGTCGA-3′
**2**	VanR$_B$_pETCH_R	5′-TTTTTTTTTTCCCGGGTAATGATTCCTCCAATCGGTAAC-3′
**3**	VanR$_B$_D53N_F	5′-GTTATTCTTAATATTATGCTGCCCGGTATGAATGGGCATGAA-3′
**4**	VanR$_B$_D53N_R	5′-CAGCATAATATTAAGAATAACCAGTTGATAGGTGTTTTCATAGAACTTG-3′
**5**	VanS$_B$_pETCH_F	5′-TTTTTTTTTTCCATGGAACGCAAAGGCATCTTCATC-3′
**6**	VanS$_B$_pETCH_R	5′-TTTTTTTTTCCCGGGCAGCGTTGAGGTCGGCG-3′
**7**	VanS$_B$_pETHSUL_F	5′-CCGCGAACAGATTGGTGGCGGTATGGAACGCAAAGGCATCTTCAT-3′
**8**	VanS$_B$_pETHSUL_R	5′-CTTCTCGAGGAGAGTTTAGACGATTACAGCGTTGAGGTCGGCGGC-3′
**9**	cVanS$_B$_pETCH_F	5′-TTTTTTTTTTCCATGGCCACGCCGATCAAAGCCCT-3′
**10**	cVanS$_B$_pETCH_R	5′-TTTTTTTTTTCCCGGGCAGCGTTGAGGTCGGCGG-3′
**11**	VanS$_B$_periplasmic_STOP_F	5′-TCAAGGTTAATGCGGTGGTAGTCAGCAGTTCGTTTCATATTTCCGTGCC-3′
**12**	VanS$_B$_periplasmic_STOP_R	5′-CCACCGCATTAACCTTGATACAGCAGGCCAACACCGGCTTTGC-3′
**13**	PhoR_Bs_fwd	5′-GAGAACCTGTACTTCCAGATGGAAACATCTGATCAAAGGAAAGCAG-3′
**14**	PhoR_Bs_rev	5′-GAAGCTTATTTAATTACCTGCATTACAATTCGCCTTTTAAGCCGTCGC-3′
**15**	PhoR_Bs_int_fwd	5′-GAATTGGGCTCTGGCGGTTCTGAAACATCTGATCAAAGGAAAGCAG-3′
**16**	PhoR_Bs_int_rev	5′-GTTTCAGAACCGCCAGAGCCCAATTCGCCTTTTAAGCCGTCGC-3′
